# Effects of the association between whole cottonseed and calcium salts of fatty acids on nutrient intake, feedlot performance, and carcass characteristics of *Bos indicus* animals offered a high-concentrate diet

**DOI:** 10.1093/tas/txab207

**Published:** 2021-10-26

**Authors:** Leonardo R Müller, Daniel I C G Gouvêa, André F Francischinelli, Guiherme D A Alvarenga, Pablo S Castagnino, Bruno I Cappellozza, Osvaldo A de Sousa, Paulo R L Meirelles, Ciniro Costa, Cyntia L Martins, Mario B Arrigoni

**Affiliations:** 1 Faculdade de Medicina Veterinária e Zootecnia, Universidade Estadual Paulista, Botucatu, SP 18618-000, Brazil; 2 Nutricorp, Araras, SP 13601-000, Brazil

**Keywords:** *Bos indicus*, calcium salts of fatty acids, isolipidic, marbling, performance, whole cottonseed

## Abstract

This experiment evaluated the effects of feeding whole cottonseed (WC) and/or calcium salts of fatty acids (CSFA) on dry matter intake (DMI), performance, and carcass characteristics of *Bos indicus* animals receiving a high-concentrate diet during the finishing phase. On day 0, 96 Nellore bulls were blocked according to initial shrunk body weight (BW; 302 ± 26.7 kg) into group pens (four animals/pen) and, within blocks, pens were randomly assigned to receive: 1) 15% of WC and 2% of CSFA (dry matter [DM] basis) of palm, cottonseed, and soybean oil (15WC; *n* = 6); 2) 10% of WC and 3% of CSFA (DM basis) of palm, cottonseed, and soybean oil (10WC; *n* = 6); 3) 5% of WC and 4% of CSFA (DM basis) of palm, cottonseed, and soybean oil (5WC; *n* = 6); and 4) 0% of WC and 5% of CSFA (DM basis) of palm, cottonseed, and soybean oil (0WC; *n* = 6). Diets were formulated to be isocaloric, isonitrogenous, and isolipidic. Experimental period lasted 108 d; DMI was evaluated daily, whereas blood samples and carcass measurements were obtained on days 0, 55, and 108 of the study. Upon slaughter on day 109, steaks were collected for determination of the chemical and fatty acid (FA) profile of the meat. No treatment effects (*P* ≥ 0.35) were observed on DMI, performance, average daily gain (ADG), carcass ultrasound measurements, and chemical variables of the steak. Nonetheless, including WC into the diets decreased C12:0, C16:0, C16:1 *trans*-9, C17:0, C18:0, C18:1 cis-9, C18:2 *cis*-9, *cis*-12, C18:3 *cis*-9, *cis*-12, *cis*-15, saturated, and unsaturated FA intake (*P* < 0.01). Moreover, adding WC increased DMI fluctuation and feed efficiency (FE; *P* = 0.03) but decreased marbling (*P* ≤ 0.03). A treatment × day interaction was observed (*P* < 0.01) for serum leptin concentration, as 10WC animals had greater leptin concentration on day 103 vs. other treatments (*P* < 0.01). Regarding steak FA profile, WC addition into the diet increased C18:2 *cis*-7, *trans-*9 and C18:3 *cis*-9, *cis*-12, *cis*-15 (*P* < 0.001), whereas saturated FA was quadratically affected (*P* = 0.02) and unsaturated FA was reduced for 15WC (*P* < 0.04). In summary, increasing levels of CSFA into isolipidic finishing diets containing WC did not negatively impact feedlot performance but reduced FE and increased marbling scores of *B. indicus* bulls, demonstrating its feasibility as a technology to improve carcass traits of low-marbling animals.

## INTRODUCTION

One of the main goals of lipid feedstuffs is to increase energy content of the diet and feed efficiency (FE) of the beef cattle herd (NASEM, 2016). In Brazil, whole cottonseed (WC) and calcium salts of fatty acids (CSFA) are the main lipid sources included in feedlot diets (Pinto and Millen, 2019). Besides WC adoption, its inclusion remains limited at 15% of dry matter (DM), given gossypol toxicity issues and potential negative effects on performance and meat sensorial traits (Cranston et al., 2006; Gouvêa et al., 2020). However, using WC as the sole lipid feedstuff might not be able to increase dietary ether extract (EE) content to values that can impact energy utilization and transfer into the carcass. As reported by Krehbiel (2014), feedlot diets containing roughly 7% EE benefit performance and carcass traits (Zinn, 1989; Zinn et al., 2000). A feasible feedstuff that could be used to increase dietary EE and improve performance of feedlot cattle is the CSFA (Carvalho et al., 2020; Nascimento et al., 2020). To the best of our knowledge, no other study evaluated the effects of levels of both WC and CSFA into feedlot cattle diets. As these differ on protection against rumen biohydrogenation, the amount and profile of fatty acids (FAs) entering the small intestine for absorption might also differ, significantly impacting metabolism and carcass traits (Jenkins et al., 2008).

*Bos indicus* are recognized as low-marbling breeds (Martins et al., 2015), and nutritional alternatives that promote marbling are warranted. In cattle, FAs inhibit or stimulate gene expression, adipocyte differentiation, and carcass characteristics (Choi et al., 2015; Costa et al., 2020). Based on this rationale, we hypothesized that varying levels of WC and CSFA would improve the performance and carcass traits of feedlot cattle. Therefore, our objective was to evaluate the effects of WC and CSFA on performance, nutrient intake, carcass traits, and meat FA profile of *B. indicus* feedlot cattle.

## MATERIALS AND METHODS

This experiment was conducted at the experimental feedlot from the Departamento de Melhoramento e Nutrição Animal, located in Botucatu, São Paulo, Brazil (22°53′25″S, 48°27′19″W, and elevation of 828 m) from April to August 2019. All animals utilized herein were cared for in accordance with acceptable practices and experimental protocols reviewed and approved by the FMVZ/UNESP Institutional Animal Care and Use Committee (CEUA #017/2019).

### Animals, Diets, and Housing

The experimental design used herein was randomized complete block design. On day 0 of the experiment, 96 non-castrated Nellore animals were blocked by initial shrunk body weight (BW = 302 ± 26.7 kg; initial age = 20 ± 2 mo) and allocated to 1 of 24 pens (four animals/pen). Within blocks (*n* = 6 blocks; 1 treatment/block), pens were randomly assigned to one of four treatments: 1) 15% of WC and 2% of CSFA (DM basis) of palm, cottonseed, and soybean oil (15WC; *n* = 6; Nutri Gordura Terminação; Nutricorp, Araras, SP, Brazil); 2) 10% of WC and 3% of CSFA (DM basis) of palm, cottonseed, and soybean oil (10WC; *n* = 6; Nutri Gordura Terminação; Nutricorp); 3) 5% of WC and 4% of CSFA (DM basis) of palm, cottonseed, and soybean oil (5WC; *n* = 6; Nutri Gordura Terminação; Nutricorp); and 4) 0% of WC and 5% of CSFA (DM basis) of palm, cottonseed, and soybean oil (0WC; *n* = 6; Nutri Gordura Terminação; Nutricorp). A complete description of the FA profile of the lipid feedstuffs (WC and CSFA) is reported in Table 1, while Tables 2 and 3 report feedstuff composition and nutrient profile of the diets offered during the adaptation, growing, and finishing periods, respectively.

**Table 1.** FA profile of the feedstuffs used in the present experiment

Fatty acids, % total FA	Corn	Corn silage	Peanut meal	CSFA	WC
C10:0 (capric acid)	0.08	0.00	0.09	0.00	0.00
C12:0 (lauric acid)	0.37	0.22	0.26	1.15	0.00
C14:0 (myristic acid)	0.33	0.41	0.24	0.96	0.76
C15:0 (pentadecanoic acid)	0.03	0.05	0.02	0.00	0.00
C16:0 (palmitic acid)	15.23	17.81	8.50	22.27	21.12
C16:1 *trans-*9 (palmitoleic acid)	0.44	0.23	0.37	1.06	0.51
C17:0 (heptadecanoic acid)	0.12	0.17	0.11	0.00	0.13
C17:1 *cis-*10 (heptadecenoic acid)	0.04	0.06	0.08	0.00	0.00
C18:0 (stearic acid)	4.10	5.15	3.19	3.77	2.41
C18:1 *cis-*9 (oleic acid)	33.81	31.87	72.73	51.58	15.54
C18:2 *cis-*9, *cis-*12 (linoleic acid)	42.02	39.87	6.73	16.13	58.72
C18:3 *cis-*9, *cis-*12, *cis-*15 (linolenic acid)	1.08	2.17	0.28	0.66	0.19
C20:0 (arachidic acid)	0.66	0.66	1.10	0.20	0.25
C20:1 (eicosenoic acid)	0.26	0.25	1.86	0.00	0.06
C22:0 (behenic acid)	0.24	0.37	2.45	0.00	0.00
C22:1 (erucic acid)	0.00	0.00	0.22	0.00	0.00
C24:0 (lignoceric acid)	0.33	0.63	1.60	0.73	0.19
Saturated FA	21.49	25.47	17.56	29.07	24.86
Unsaturated FA	78.38	74.45	82.45	69.44	75.02
Monounsaturated	0.48	0.29	0.67	1.06	0.51
Polyunsaturated	43.66	42.04	7.05	16.79	58.91

**Table 2.** Composition (DM basis) of the adaptation (ADAP), growing (GRO), and finishing (FIN) diets offered during the present experiment^†,‡^

Item, % DM	ADAP-1		ADAP-2		ADAP-3		GRO				FIN			
	5WC	0WC	5WC	0WC	5WC	0WC	15WC	10WC	5WC	0WC	15WC	10WC	5WC	0WC
Corn silage	40.0	40.0	35.0	35.0	28.0	28.0	14.0	15.0	22.0	27.0	10.0	13.0	16.0	16.0
Ground corn	34.4	37.0	40.5	42.7	46.4	48.4	64.3	66.0	60.5	58.0	69.0	68.4	67.2	69.0
Peanut meal	15.5	17.1	14.5	16.3	14.5	16.5	1.2	2.5	5.0	6.5	1.0	2.5	4.5	5.4
Whole cottonseed	5.0	—	5.0	—	5.0	—	15.0	10.0	5.0	—	15.0	10.0	5.0	—
CSFA	1.6	2.6	1.6	2.6	2.7	3.7	2.0	3.0	4.0	5.0	1.5	2.6	3.8	5.0
Urea	0.9	0.9	0.9	0.9	0.9	0.9	1.0	1.1	1.1	1.1	1.0	1.1	1.1	1.1
Mineral–vitamin mix^||^	2.5	2.5	2.5	2.5	2.5	2.5	2.5	2.5	2.5	2.5	2.5	2.5	2.5	2.5

ADAP-1, adaptation diet 1 offered for from day 0 to 5; ADAP-2: adaptation diet 2 offered for from day 6 to 10; ADAP-3, adaptation diet 3 offered for from day 11 to 15; GRO, growing diet; FIN, finishing diet.

^†^Experimental period lasted 108 d.

^‡^Nutritonal profile and composition of the adaptation diets were the same for treatments 5WC, 10WC, and 15WC in order to adapt animals to the increasing level of EE into the diet.

^||^Mineral–vitamin mix contained 22% Ca, 2.6% S, 2% Mg, 8,5% Na, 2.0% P, 200 ppm Zn, 142 ppm Mn, 533 ppm Cu, 10.8 ppm, 40 ppm Co, and 1,000 ppm monensin.

**Table 3.** Nutritional profile (DM basis) of the adaptation (ADAP), growing (GRO), and finishing (FIN) diets offered during the present experiment^†,‡^

Item, % DM	ADAP-1		ADAP-2		ADAP-3		GRO				FIN			
	5WC	0WC	5WC	0WC	5WC	0WC	15WC	10WC	5WC	0WC	15WC	10WC	5WC	0WC
Nutritional profile														
CP	19.5	19.5	19.3	19.3	19.3	19.3	15.5	15.5	15.5	15.5	15.5	15.5	15.5	15.5
EE	6.0	6.0	6.0	6.0	6.8	6.8	7.5	7.5	7.5	7.5	7.5	7.5	7.5	7.5
NDF	30.0	27.6	28.5	26.1	26.5	24.2	24.9	22.7	22.4	21.6	23.6	22.2	20.7	20.3
Physically effective NDF	27.0	24.0	25.0	23.0	23.0	21.0	24.0	21.0	21.0	19.0	23.0	21.0	19.0	18.0
Starch	42.0	44.0	44.0	46.0	45.0	47.0	51.0	53.0	53.0	53.0	53.0	54.0	54.0	54.0
TDNs^||^	75.0	77.0	76.0	78.0	78.0	80.0	78.0	80.0	80.0	82.0	79.0	80.0	82.0	83.0
Metabolizable energy^$^, Mcal/kg	2.72	2.77	2.74	2.80	2.81	2.87	2.83	2.89	2.92	2.96	2.84	2.89	2.95	2.99
NE for maintenance^$^, Mcal/kg	1.79	1.84	1.81	1.87	1.87	1.93	1.89	1.94	1.96	2.00	1.89	1.94	1.99	2.02
NE for gain^$^, Mcal/kg	1.17	1.21	1.18	1.23	1.24	1.28	1.24	1.28	1.31	1.34	1.27	1.31	1.35	1.37
FA, % total FA														
C16:0 (palmitic acid)	11.4	4.7	11.5	4.6	13.2	5.3	27.9	26.8	24.7	22.4	27.0	26.2	25.9	23.7
C16:1 *trans-*9 (palmitoleic acid)	0.4	0.0	0.4	0.0	0.5	0.0	0.1	0.3	0.2	0.4	0.2	0.4	0.2	0.4
C18:0 (stearic acid)	2.3	2.8	2.3	2.8	2.6	3.2	4.9	4.5	4.0	3.8	4.4	4.2	4.2	3.8
C18:1 *cis-*9 (oleic acid)	25.4	35.3	25.3	35.3	30.0	40.4	37.5	34.5	29.5	26.0	34.4	32.5	31.3	28.5
C18:2 *cis-*9, *cis-*12 (linoleic acid)	22.9	28.1	23.4	28.9	24.0	29.7	24.5	29.1	38.1	44.8	29.4	33.1	34.2	40.2
C18:3 *cis-*9, *cis-*12, *cis-*15 (linolenic acid)	0.5	0.1	0.5	0.1	0.5	0.1	0.8	0.7	0.6	0.5	0.6	0.5	0.9	0.5
Saturated FA	15.5	17.9	15.7	18.0	17.9	20.7	36.0	34.2	30.9	28.0	34.3	32.9	32.7	30.0
Monounsaturated FA	24.7	23.2	25.1	23.3	26.3	27.8	38.2	35.2	30.3	26.5	35.1	33.2	31.9	29.2
Polyunsaturated FA	23.4	16.8	24.0	17.3	24.6	19.2	25.7	30.4	38.8	45.5	30.5	33.9	35.4	41.0

ADAP-1, adaptation diet 1 offered for from day 0 to 5; ADAP-2, adaptation diet 2 offered for from day 6 to 10; ADAP-3, adaptation diet 3 offered for from day 11 to 15; GRO, growing diet; FIN, finishing diet.

^†^Experimental period lasted 108 d.

^‡^Nutritonal profile and composition of the adaptation diets were the same for treatments 5WC, 10WC, and 15WC in order to adapt animals to the increasing level of EE into the diet.

^||^Calculated according to equations described by Weiss et al. (1992).

^$^Calculated according to equations described by NASEM (2016).

Given that the overall objective of this experiment was to evaluate the associative effect of WC and CSFA, all diets were formulated to be isolipidic, isonitrogenous, and isocaloric (Table 3). The experimental period lasted 108 d, whereas the adaptation, growing, and finishing diets were offered from days 0 to 15, 16 to 55, and 56 to 108, respectively. During the adaptation diet, all animals were allocated to *step-up* diets for a 15-d period, in which roughage amount was reduced from 40% to 28% (DM basis) and EE amount gradually increased from 6.0% to 6.8% (DM basis), as reported in Table 2. All diets were formulated using the Large Ruminant Nutrition System (Fox et al., 2004) and formulated to result in an average daily gain (ADG) of approximately 1.7 kg/d. Diets were offered twice a day (0900 and 1600 h), at a rate of 55:45% of the total amount, respectively, and all animals had ad libitum access to water and the diets to result in 2% to 3% orts. Throughout the experimental period, all animals were maintained in a roofed barn with concrete-based floors (7.5 m^2^/animal and 1.25 m of linear feedbunk/animal).

Seven days prior to the beginning of the experiment, all animals were dewormed (Dectomax; Zoetis Saúde Animal, São Paulo, SP, Brazil) and vaccinated against *Clostridium* (Covexin-10; MSD Saúde Animal, São Paulo, SP, Brazil). Moreover, from day −7 to −1 of the experimental period, all animals received a diet containing (DM basis) 35% sugarcane bagasse, 20% corn silage, 25% ground corn, 18% peanut meal, 0.5% urea, and 1.5% mineral–vitamin mix. Diets were offered at a rate of 2% BW obtained at feedlot facility arrival (day –7). This management was performed to provide similar substrates and potentially equalize the rumen microbiome profile of the animals prior to treatment administration.

### Body Weight, Feed, and Blood Sampling

Individual animal shrunk BW was collected at the beginning (day 0) and end of the experimental period (day 108) after 16 h of feed and water withdrawal. With these BW measurements, BW change (final minus initial BW) and overall ADG during the experiment were calculated and further analyzed. Additional full BW measurements were taken at the end of each dietary management (adaptation and growing diets on day 15 and 55, respectively), in order to follow up herd performance as the experiment progressed. Intermediate full BW, instead of shrunk BW, was obtained to prevent extreme dry matter intake (DMI) fluctuations following management and realimentation that could result in any potential rumen disorders (Allen et al., 2009) and to avoid the trigger of an acute-phase response that feed withdrawal might cause on the animals and subsequently impair feedlot performance and health (Marques et al., 2012, 2019).

Throughout the experimental period, total DMI was evaluated daily from each pen by collecting and weighing refusals. Samples of the offered and nonconsumed feed were collected daily from each pen and dried for 96 h at 50 °C in forced-air ovens for DM determination. The total DMI of each pen was divided by the number of animals within each pen and expressed as kg/animal per day. In addition, total DMI was expressed in percentage of BW by dividing the average DMI and average BW (initial + final) of each pen during the experimental period. At the end of the experimental period (day 108), FE was calculated by dividing ADG and DMI, whereas DMI fluctuation was estimated by weighing diet offered and orts for actual DMI and fluctuation calculated as the difference between DMI on two consecutive days (Bevans et al., 2005). 

Calculation of ration total digestible nutrients (TDNs) concentration was performed according to equations proposed by Weiss et al. (1992), and samples were analyzed in duplicates by wet chemistry procedures for concentrations of crude protein (CP), EE, and ash (AOAC, 1990). Neutral detergent fiber (NDF) was evaluated according to procedures described by Van Soest et al. (1991). Moreover, digestible energy (DE) and metabolizable energy (ME) were performed according to equations described by NASEM (2016), whereas dietary calculations of net energy for maintenance (NE_m_) and gain (NE_g_) followed equations proposed by Zinn et al. (2002). For these equations, retained energy (RE) for a high-frame 18-mo animal was: RE = [0.0437 × BW^0.75^] × ADG × 1.097 (NRC, 1984), where BW = mean BW minus 4% for intermediate values only. Net energy for gain was determined through the estimation reported by Zinn and Shen (1998): NE_g_ = NE_m_ × 0.877 − 0.41. Additionally, all feedstuffs used in the present experiment (Table 1) were analyzed for FA profile following the procedures described in AOCS (2009), and according to total DMI, individual and total saturated, monounsaturated, and polyunsaturated FA intakes were calculated and estimated for each treatment and subsequently reported herein.

On days 0, 55, and 103 of the experimental period, blood samples were collected for plasma concentrations of leptin and lactate. All blood samples were collected via jugular venipuncture into commercial blood collection tubes (Vacutainer, 10 mL; Becton Dickinson, Franklin Lakes, NJ). After collection, blood samples were centrifuged at 3,000 rpm for 15 min, and one sample stored at −20 °C for subsequent plasma leptin analyses. Plasma leptin concentration was determined by using a commercial radioimmunoassay ^125^I kit (Linco Research, Inc., St. Charles, MO, USA), following the procedures reported by Pelleymounter et al. (1995). The second blood sample was immediately used for lactate determination by using a colorimetric reagent test (Accutrend; F. Hoffmann-La Roche Ltd., Rotkreuz, Switzerland).

### Dietary Particle Size Classification

The classification of the particle size of the diets was performed by using the *Penn State Particle Separator* (PSPS; Heinrichs, 2013), and the not accounted/predicted intake ratio was taken into consideration herein, where *n* = 19 (long), 8 (medium), 1.18 mm (small), and bottom (fine) sieves. Values obtained for this classification include 1 = no classification (null), <1 = selective intake refusal (negative ratio), and >1 = preferential intake (positive ratio). Corn silage particle size and WC were also determined by PSPS and had, on average, 9.30 and 11.96 mm, respectively, whereas for other feedstuffs, a different set of sieves was used (6.0, 3.25, 2.0, 1.25 mm, and bottom). Particle size of the concentrates was determined using the methodology described by ASABE (2008). The average particle size of corn, peanut meal, CSFA, urea, and mineral–vitamin mix was 3.60, 2.40, 1.05, 2.30, and 0.73 mm, respectively.

### Ultrasound Evaluation

On days 0, 55, and 103 of the experimental period, all animals enrolled into the experiment were submitted to ultrasound evaluations (Aloka SSD-500V with a 17.2 cm/3.50 MHz convex probe; Hitachi Healthcare Americas, Twinsburg, OH), and evaluations were performed by the same trained technician (DGT Brasil, Presidente Prudente, SP, Brazil). Evaluations were conducted according to procedures described by the Ultrasound Guidelines Council (UGC, 2012), and measurements of the ribeye area (REA; cm^2^), marbling (%), and backfat thickness (BFT; mm) were collected on the longissimus muscle between the 12th and 13th ribs and from the biceps femoris muscle. Additionally, overall REA, marbling, and BFT gains were calculated as delta (Δ; final measurement − initial measurement) and the Δ was divided by the number of days between experimental measurements (103 d) to represent the daily gain of these parameters (cm^2^ for REA, % for marbling, and mm for BFT of both tissues).

### Carcass Measurements

At the end of the experimental period, all animals were slaughtered on day 109 following a waiting period of approximately 16 h, in a federally inspected commercial packing plant (Frigol, Lençóis Paulista, SP, Brazil). Hot carcasses were separated into two symmetrical sections, weighed to obtain hot carcass weight (HCW), and individually identified. Dressing percent (DP) was calculated by dividing the HCW by final shrunk BW of the animal on day 108 of the experiment. Per standard industry procedures, initial DP of the animals was estimated at 50% (Costa et al., 2020) and then it was calculated the amount of carcass gained by the animals during the experimental period (day 0 to 108). Carcass ADG was calculated by dividing the carcass gain and the number of days on feed (108 d), whereas yield gain (YG) was calculated by dividing carcass ADG (in kg) and ADG (in kg).

### Meat Traits Evaluations

Following slaughter, all carcasses were cooled in a cold chamber for 48 h, whereas samples of longissimus muscle from the left carcasses were collected for further laboratorial analyses. For the colorimetric, weight losses due to cooking, and shear force analysis, three pieces of 2.54 cm thickness each were collected proximally to the 12th and 13th ribs. All pieces were individually identified, vacuum-packed, and stored (−20 °C) until further laboratorial analysis.

#### Proximate composition

. Meat moisture, CP, EE, and ash content were evaluated in samples collected from longissimus muscles. Moisture and CP contents were determined according to the methodologies previously described by AOAC (2007; methods 950.46 and 981.10, respectively). Ether extract and ash contents were also determined according to methods 920.39 and 920.153 reported in AOAC (2007).

#### Colorimetric, pH, weight loss due to cooking, and shear force analysis

. Upon analysis, samples were thawed in a refrigerator for 24 h and exposed to oxygen (O_2_) for 20 min. Following this procedure, meat pH was determined using a digital device (model HI-99163; Hanna Instruments Brasil, Barueri, SP, Brazil). For colorimetric parameters, the determination of the components L*, a*, and b* was performed according to Abularach et al. (1998). Color was evaluated on the surface of the samples using the CIE L*, a*, and b* system with a D65 illuminating and 10° as the standard evaluation point. A Minolta CM-2500-D device (Konica Minolta, Osaka, Japan) was used for color determination and calibrated with a blank standard sample. The following color parameters were used: L* is a lightness index (0 = black and 100 = white), a* is the intensity of red color, an index that ranges from green (−) to red (+), and b* is the intensity of yellow color, an index ranging from blue (−) to yellow (+; Houben et al., 2000).

Following color measurement, longissimus samples were identified, weighed in a high-precision scale, and cooked at 170 °C until internal temperature reached 71 °C, as described by the American Meat Science Association (AMSA, 1995). Then, samples were cooled at room temperature and weighed in order to determine the weight loss due to cooking (final − initial weight; Honikel, 1998). The same samples were subsequently enveloped in a plastic film and placed on the refrigerator (4 to 6 °C) for 24 h. After 24 h, 6 to 8 cylinders of meat were collected from each sample (1.27 cm diameter each), parallel to the longitudinal orientation of the muscle fibers. For shear force determination, the cylinders were sheared in a Warner–Bratzler Shear Force (WBSF) device with a 20 cm/min velocity, whereas shear force of each sample was determined as the average of the 6 to 8 replicates and expressed as kilogram (Wheeler et al., 1997).

#### Meat FA profile

. A subsample (approximately 2.8 g) of the longissimus muscle was used by adding it to a 50-mL Falcon tube (CRAL, Cotia, SP, Brazil). The extraction procedure followed the methodology described by Folch et al. (1957). Briefly, lipids were extracted by homogenizing the sample with a 2:1 chloroform:methanol solution (Ultra Turrax; Marconi Equipamentos, Piracicaba, SP, Brazil) and isolated after the addition of a 1.5% NaCl solution. The isolated lipid was methylated and methyl esters were assembled according to the methodology described by Kramer et al. (1997). Fatty acids were quantified by gas chromatography (GC-2010 Plus Capillary GC; Shimadzu Scientific Instruments Inc., Columbia, MD) using an SP-2560 capillary column (100 m × 0.25 mm of diameter and 0.02 mm thickness; Supelco Inc., Bellefonte, PA). Initial column temperature was 45 °C with a progressive heating until temperature reached 175 °C, which was kept for 27 min. Then, temperature was increased at a rate of 4 °C/min until 215 °C, which was kept for 35 min. Hydrogen gas was used as the carrier with a 40 cm^3^/s flux. Fatty acids were identified using previously known and validated standards (methyl tricosanoate; Sigma-Aldrich, Saint Louis, MO), quantified by evaluating the retention time of the samples and normalization of the peak area of methyl esters with the Chromquest 5.0 Clarity Lite software (version 2.4.191; DataApex Software, Prague, Czech Republic), whereas all FAs were expressed as mg/g of total FA.

### Statistical Analysis

For all analyses performed herein, pen was considered the experimental unit. All data were analyzed using the MIXED procedure of SAS (version 9.4; SAS Inst. Inc.; Cary, NC, USA) and the Satterthwaite approximation to determine the denominator df for the test of fixed effects. The model statement used for all analyses contained the fixed effects of treatment. All data, with the exception of DMI and FE, were analyzed using block, animal(pen), and pen(treatment) as random variables. For DMI and FE, block and pen(treatment) were used as random variables. Blood samples and ultrassound measurements obtained on day 0 were analyzed for covariates, and results obtained on days 55 and 103 were covariately adjusted. Moreover, DMI (kg and % BW), DMI fluctuation, particle classification, blood, and ultrasound data were analyzed as repeated variables, containing day in the repeated statement, the subject was pen(treatment), and the covariance structure was first-order autoregressive, which provided the best fit for these analyses according to the smallest Akaike Information Criterion and Schwarz Bayesian Criterion.

For specific mean analysis comparison, orthogonal contrasts were used to partition specific treatment effects, given that the contrast 1) compared the inclusion of WC: 0WC vs. WC, 2) linear contrast, and 3) quadratic contrast. For all data, means were separated using the LSMEANS, significance was set at *P* ≤ 0.05, and tendencies were denoted if 0.05 < *P* ≤ 0.10.

## RESULTS

### Intake, Performance, and Blood Profile

As expected, no treatment effects were observed on initial BW (*P* ≥ 0.90), indicating that animals were managed similarly prior to the beginning of the experiment (Table 4). At the end of the experimental period, BW, DMI, and DMI as percentage of BW did not differ among treatments (*P* ≥ 0.17). Nonetheless, ADG and FE were improved (*P* ≤ 0.02) during the adaptation period (day 0 to 15) and overall (day 0 to 108) FE also was improved (*P* = 0.03) when WC was included in the diets, whereas overall ADG was not impacted by the experimental treatments offered herein (*P* ≥ 0.42). Additionally, removing WC of the diets significantly reduced (*P* = 0.03) DMI fluctuation, but no linear or quadratic effects were observed on this parameter (*P* ≥ 0.13).

**Table 4.** Feedlot performance of *Bos indicus* bulls offered isolipidic diets containing 15%, 10%, 5%, or 0%(DM basis) of WC and 2%, 3%, 4%, or 5% of CSFA as lipid sources^†^

Item^‡^	Treatments				SEM	*P*-value		
	15WC	10WC	5WC	0WC		0WC vs. WC	L	Q
BW, kg								
Initial	302.7	302.2	300.7	303.0	10.92	0.93	0.99	0.90
Day 15	324.6	328.7	336.5	335.4	11.55	0.69	0.44	0.82
Day 55	387.0	389.5	390.2	393.9	13.72	0.77	0.75	0.94
Final	478.3	485.2	481.1	485.5	16.84	0.84	0.82	0.94
ADG, kg								
Days 0 to 15	2.04	2.21	2.25	1.75	0.15	0.02	—	—
Days 16 to 55	1.47	1.45	1.43	1.49	0.08	0.67	0.89	0.66
Days 56 to 108	1.79	1.83	1.76	1.74	0.07	0.51	0.49	0.68
Overall	1.68	1.72	1.68	1.63	0.09	0.42	0.54	0.52
DMI, kg/d								
Days 0 to 15	9.21	9.26	9.43	9.21	0.33	0.80	—	—
Days 16 to 55	10.79	11.65	10.97	11.30	0.41	0.73	0.65	0.54
Days 56 to 108	11.95	11.94	11.45	11.70	0.47	0.89	0.56	0.77
Overall	11.16	11.48	10.98	11.21	0.41	0.99	0.85	0.90
DMI, % BW								
Days 0 to 15	2.92	2.97	3.01	2.91	0.06	0.42	—	—
Days 16 to 55	3.00	3.24	3.05	3.14	0.05	0.45	0.30	0.17
Days 56 to 108	2.74	2.73	2.62	2.69	0.05	0.98	0.31	0.41
Overall	2.86	2.95	2.84	2.90	0.05	0.86	0.93	0.72
FE, g/kg								
Days 0 to 15	220	238	236	191	10	<0.01	—	—
Days 16 to 55	135	124	130	132	4	0.70	0.76	0.11
Days 56 to 108	148	152	154	148	3	0.35	0.82	0.08
Overall	150	149	153	145	2	0.03	0.26	0.12
DMI fluctuation^||^,%								
Days 0 to 15	6.56	6.44	5.82	5.66	0.69	0.44	—	—
Days 16 to 55	3.95	3.24	5.65	3.32	0.45	0.06	0.81	0.07
Days 56 to 108	3.10	2.69	3.43	2.46	0.35	0.12	0.45	0.42
Overall	3.90	3.53	4.50	3.29	0.28	0.03	0.48	0.13

L, linear effect; Q, quadratic effect.

^†^Diets were offered for 108 d.

^‡^Body weight measurements on days 15 and 55 were obtained as full BW, whereas measurements obtained on days 0 and 108 were obtained from animals that were restricted from feed and water for 16 h.

^||^Calculated according to methodologies described by Bevans et al. (2005).

In agreement with the DMI data, no contrast effects were observed for any of the estimated nutrient intakes analyzed herein (*P* ≥ 0.14; Table 5). Adding WC in the diets caused a reduction in intakes of C12:0; C16:0; C17:0; C18:0; C18:1 *cis*-9; C18:3 *cis-*9, *cis-*12, *cis-*15; C20:0; C20:3 *n*-6; C24:0; saturated (SFA); and monounsaturated (MUFA) FA, as well as SFA:polyunsaturated FA (PUFA) ratio (*P* ≤ 0.05; Table 5). On the other hand, intakes of C16:1 *trans*-9, C18:2 cis-9, *cis*-12, C23:0, unsaturated FA (UFA) and PUFA, as well as SFA:MUFA ratio were increased by WC inclusion (*P* ≤ 0.01; Table 5). Additionally, with the exception of C17:0, all FA intakes were linearly impacted by the treatments (*P* ≤ 0.01), whereas C14:0; C17:0; C18:3 *cis-*9, *cis-*12, *cis-*15; C20:1 *n*-9; C23:0; and C24:0 FA intakes were affected in a quadratic fashion (*P* ≤ 0.05; Table 5).

**Table 5.** Nutrient intake of *Bos indicus* bulls offered isolipidic diets containing 15%, 10%, 5%, or 0% (DM basis) of WC and 2%, 3%, 4%, or 5% of CSFA as lipid sources^†^

Item	Treatments				SEM	*P*-value		
	15WC	10WC	5WC	0WC		0WC vs. WC	L	Q
Nutrient intake, kg/d								
CP	1.74	1.84	1.81	1.75	0.06	0.53	0.92	0.16
EE	0.80	0.85	0.94	0.81	0.03	0.54	0.90	0.17
NDF	2.50	2.61	2.61	2.53	0.10	0.73	0.83	0.38
Physically effective NDF	2.30	2.39	2.41	2.35	0.11	0.91	0.75	0.54
Starch	5.67	6.07	5.93	5.74	0.19	0.50	0.94	0.14
TDN	8.74	9.14	8.85	9.20	0.33	0.45	0.47	0.94
FA intake, g/d								
C12:0 (lauric acid)	2.31	2.92	3.40	3.99	0.14	<0.01	<0.01	0.94
C14:0 (myristic acid)	4.61	5.19	5.67	5.38	0.17	0.29	<0.01	0.02
C16:0 (palmitic acid)	194.1	219.2	218.1	227.8	7.20	0.05	<0.01	0.29
C16:1*trans-*9 (palmitoleic acid)	3.46	2.28	2.90	1.90	0.10	<0.01	<0.01	0.36
C17:0 (heptadecanoic acid)	1.82	1.14	1.14	1.70	0.07	<0.01	0.24	<0.01
C18:0 (stearic acid)	31.84	35.58	35.91	38.55	1.21	<0.01	<0.01	0.65
C18:1 *cis-*9 (oleic acid)	229.1	263.1	274.9	292.8	8.61	<0.01	<0.01	0.36
C18:2 *cis-*9, *cis-*12 (linoleic acid)	352.3	308.6	259.4	242.8	11.68	<0.01	<0.01	0.26
C18:3 *cis-*9, *cis-*12, *cis-*15 (linoleinic acid)	4.62	6.48	5.05	6.10	0.19	<0.01	<0.01	0.04
C20:0 (arachidic acid)	4.13	4.55	4.54	5.27	0.17	<0.01	<0.01	0.36
C20:1 (eicosenoic acid)	1.82	2.28	2.78	2.38	0.08	0.34	<0.01	<0.01
C20:3 *n*-6 (dihomo-gamma-linolenic acid)	1.82	1.79	2.27	2.30	0.08	<0.01	<0.01	0.68
C23:0 (tricosanoic acid)	1.82	1.14	1.14	1.19	0.05	<0.01	<0.01	<0.01
C24:0 (lignoceric acid)	3.46	5.20	4.54	5.38	0.16	<0.01	<0.01	<0.01
Saturated FA	243.8	275.1	275.6	289.8	9.99	0.04	0.01	0.40
Monounsaturated FA	234.9	269.3	281.1	298.8	9.4	<0.01	<0.01	0.38
Polyunsaturated FA	358.7	316.8	267.7	252.2	11.85	<0.01	<0.01	0.28
Total FA	837.4	873.5	824.4	840.8	10.41	<0.01	<0.01	0.75
Ratio								
SFA:MUFA	1.03	1.02	0.98	0.97	0.005	<0.01	< 0.01	0.53
SFA:PUFA	0.68	0.86	1.03	1.17	0.029	<0.01	< 0.01	0.38

L, linear effect; Q, quadratic effect.

^†^Diets were offered for 108 d. Total DMI was evaluated daily throughout the experimental period, and intake was calculated as the difference between offer and orts in the following day.

Blood samples collected on day 0 were not significant covariates (*P* = 0.31) for serum lactate concentrations and did not differ among treatments (*P* = 0.67; 5.55, 6.51, 5.15, and 5.38 mMol/L for 15WC, 10WC, 5WC, and 0WC, respectively; SEM = 0.872). No significant treatment effects were observed on serum lactate concentrations, regardless of whether the data were analyzed as repeated (*P* ≥ 0.72) or as orthogonal contrasts (*P* ≥ 0.38; 4.19, 3.55, 3.54, and 3.88 mMol/L for 15WC, 10WC, 5WC, and 0WC, respectively; SEM = 0.506). Nonetheless, a day effect was observed for serum lactate (*P* = 0.02), as concentrations on day 103 were greater than values on day 55 (3.36 vs. 4.29 mMol/L, respectively).

**Figure 1.**
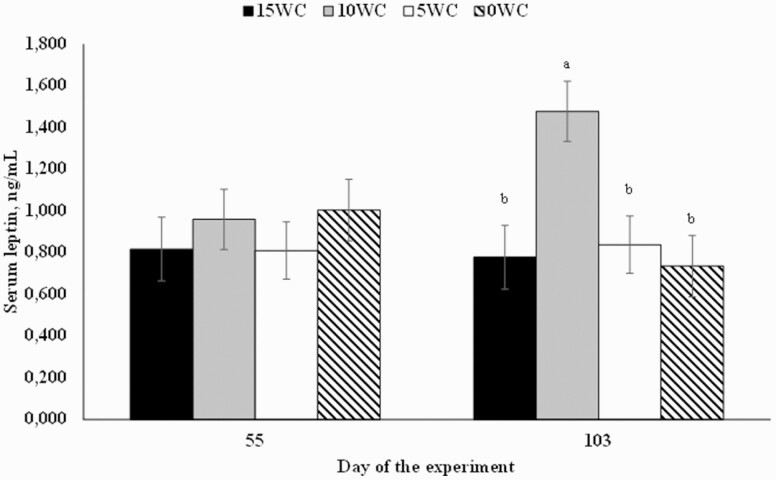
Serum concentration of leptin in *Bos indicus* bulls offered isolipidic diets containing 15%, 10%, 5%, or 0% (DM basis) of WC and 2%, 3%, 4%, or 5% of CSFA as lipid sources. A treatment × day interaction was observed on serum lactate (*P* < 0.01). Different letters indicate differences at *P* < 0.05 level.

Conversely, leptin concentrations on day 0 were significant covariates (*P* ≤ 0.03) and also did not differ among treatments (*P* = 0.37; 0.74, 0.69, 0.75, and 0.57 ng/mL for 15WC, 10WC, 5WC, and 0WC, respectively; SEM = 0.083). A treatment × day interaction was detected on serum concentration of leptin (*P* < 0.01; Fig. 1). On day 103 of the study, 10WC-fed animals had a greater serum leptin concentration compared with all other treatments (*P* < 0.01), and no further differences were observed among the other treatments (*P* ≥ 0.36; Fig. 1). Therefore, a quadratic effect was observed on mean leptin concentration (*P* = 0.02), as 10WC bulls had a greater mean serum concentration vs. 15WC and 5WC (*P* ≤ 0.04) and tended to have a greater mean serum leptin vs. 0WC (*P* = 0.07; data not shown).

### Efficiency of Dietary Net Energy Utilization

From day 0 to 15, both NE_m_ and NE_g_ increased by adding WC in the experimental diets (*P* < 0.01; Table 6), but no further differences were observed in the other periods and/or when the entire experimental period was evaluated (day 0 to 108; *P* ≥ 0.12; Table 6). Nonetheless, the same effect (0WC vs. WC) was detected on the observed:expected ratio of NE_m_ and NE_g_ from day 0 to 15 and from day 56 to 108 (*P* ≤ 0.01; Table 6), in a manner that adding WC improved these ratios from day 0 to 15 and from day 56 to 108. Additionally, observed:expected ratio of NE_m_ and NE_g_ linearly increased as WC inclusion in the diets increased (*P* ≤ 0.02; Table 6).

**Table 6.** Dietary NE utilization efficiency of *Bos indicus* bulls offered isolipidic diets containing 15%, 10%, 5%, or 0% (DM basis) of WC and 2%, 3%, 4%, or 5% of CSFA as lipid sources^†^

Item	Treatments				SEM	*P*-value		
	15WC	10WC	5WC	0WC		0WC vs. WC	L	Q
NE_m_, Mcal/kg								
Days 0 to 15	2.22	2.33	2.32	2.01	0.085	0.01	—	—
Days 16 to 55	1.71	1.61	1.67	1.66	0.034	0.94	0.54	0.17
Days 56 to 108	1.99	2.04	2.05	1.99	0.031	0.32	0.86	0.08
Overall	1.91	1.90	1.93	1.86	0.028	0.12	0.41	0.30
NE_g_, Mcal/kg								
Days 0 to 15	1.53	1.63	1.63	1.35	0.075	0.01	—	—
Days 16 to 55	1.09	1.05	1.05	1.05	0.03	0.94	0.54	0.17
Days 56 to 108	1.33	1.38	1.39	1.34	0.027	0.32	0.87	0.08
Overall	1.26	1.25	1.28	1.22	0.024	0.12	0.40	0.29
Observed:expected ratio								
NE_m_								
Days 0 to 15	1.22	1.28	1.18	1.06	0.046	0.01	—	—
Days 16 to 55	0.91	0.83	0.85	0.83	0.017	0.12	0.01	0.13
Days 56 to 108	1.05	1.05	1.03	0.99	0.016	<0.01	0.01	0.18
NE_g_								
Days 0 to 15	1.28	1.36	1.35	1.08	0.068	0.01	—	—
Days 16 to 55	0.88	0.78	0.80	0.78	0.025	0.17	0.02	0.02
Days 56 to 108	1.05	1.05	1.03	0.97	0.020	0.01	0.01	0.19

L, linear effect; Q, quadratic effect.

^†^Diets were offered for 108 d. Total DMI was evaluated daily throughout the experimental period and intake was calculated as the difference between offer and orts on the following day.

### Dietary Selectivity Index

**Figure 2.**
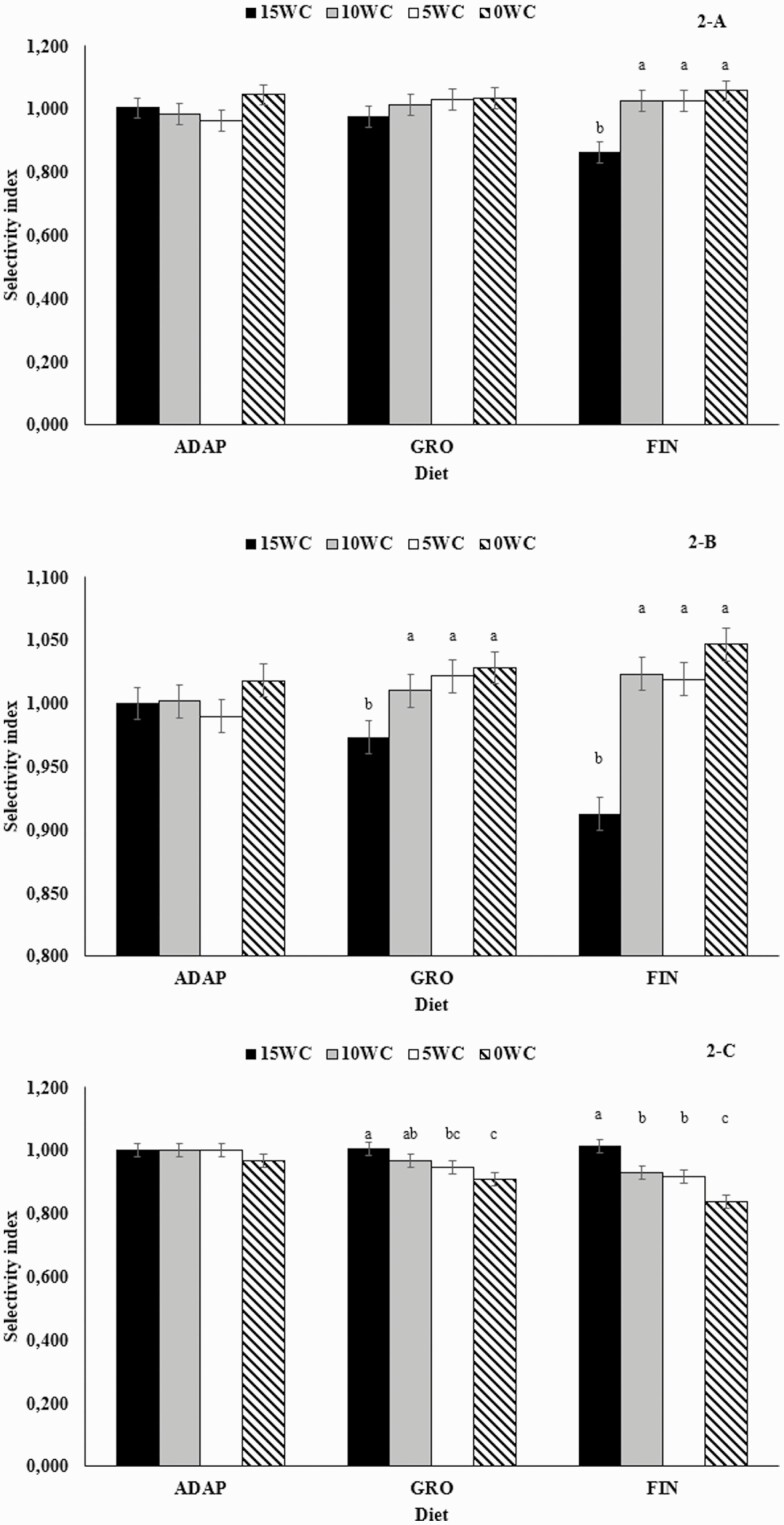
Dietary selectivity index of *Bos indicus* bulls offered isolipidic diets containing 15%, 10%, 5%, or 0% (DM basis) of WC and 2%, 3%, 4%, or 5% of CSFA as lipid sources. Treatment × diet interactions were observed for long (A), medium (B), and fine (C) particles (*P* ≤ 0.03). ADAP, adaptation diet; GRO, growing diet; FIN, finishing diet.

Treatment × diet interactions were significant for long, medium, and fine particles (*P* ≤ 0.03), whereas the same interaction tended to be observed for small particles (*P* = 0.07; Table 7 and Fig. 2). Animals fed 15WC consumed not only a reduced amount of long-sized particles in the period which the finishing diet (from d 56 to 108) was offered vs. 0WC, 5WC, and 10WC but also a reduced amount of medium-sized particles from when both the growing and finishing diets were fed when compared with other treatments (*P* ≤ 0.03; Fig. 2A and B). Conversely, intake of fine particles was not only greater for 15WC vs. 5WC and 0WC during the feeding of the growing and finishing diets (*P* < 0.01) but also greater vs. 10WC when the finishing diet was fed (*P* < 0.05; Fig. 2C). Additionally, 10WC had a greater intake of fine particles vs. 0WC during the growing and finishing diets and 5WC also had a greater intake of these particles vs. 0WC during the finishing diet (*P* < 0.01; Fig. 2C).

**Table 7.** Dietary selectivity index of *Bos indicus* bulls offered isolipidic diets containing 15%, 10%, 5%, or 0% (DM basis) of WC and 2%, 3%, 4%, or 5% of CSFA as lipid sources^†^

Item	Treatments				SEM	*P*-value^‡^		
	15WC	10WC	5WC	0WC		T	D	T × D
Particle selectivity								
19 mm (long)	0.947^b^	1.008^a^	1.008^a^	1.046^a^	0.019	0.01^*^	0.55	0.03
8 mm (medium)	0.962^b^	1.012^a^	1.010^a^	1.031^a^	0.009	<0.01^*^	0.49	<0.01
1.18 mm (small)	1.008^a^	1.001^b^	1.000^b^	1.001^b^	0.002	0.03^**^	<0.01	0.08
Bottom (fine)	1.006^a^	0.965^b^	0.955^b^	0.905^b^	0.015	<0.001^*^	<0.01	0.02

T, treatment effect; D, diet (adaptation, growing, or finishing) effect; T × D, treatment × diet interaction.

^†^Diets were offered for 108 d. Total DMI was evaluated daily throughout the experimental period and intake was calculated as the difference between offer and orts on the following day.

^‡^Symbols in *P*-value for treatment also denote a linear (^*^) and quadratic (^**^) effect in the orthogonal contrast analysis.

^a,b^Different letters in the same line indicate differences at the *P* < 0.05 level.

Additional treatment effects were observed for all particle sizes (*P* ≤ 0.03) evaluated herein (Table 7), as well as linear and quadratic effects in the orthogonal contrast analysis. Adding WC linearly decreased intake of long- and medium-sized particles (*P* ≤ 0.05), whereas the opposite was observed for fine particles (*P* = 0.02; Table 7). The quadratic effect observed herein for small particles is related to the reduced intake on 5WC and greater intake on 15WC (*P* = 0.05; Table 7).

### Ultrasound Measurements and Carcass Characteristics

Values obtained on day 0 of ultrasound measurements were significant covariates (*P* < 0.01) but did not differ among treatments (*P* ≥ 0.41; Table 8). No significant effects were observed for REA, longissimus and biceps femoris BFT (*P* ≥ 0.16), whereas the only exception to that is the linear effect on biceps femoris BFT evaluated on day 55, which decreased as WC content of the diet increased (*P* = 0.04; Table 8). Animals fed 0WC had a greater marbling content vs. WC on days 55 and 103 of the study (*P* ≤ 0.01) as well as a greater delta (final − initial marbling) score (*P* = 0.02; Table 8). A linear effect was also detected for the aforementioned variables, in a manner that marbling decreased as WC content of the diet increased (*P* = 0.01; Table 8).

**Table 8.** Ultrasound measurements (longissimus and biceps femoris) and carcass characteristics of *Bos indicus* bulls offered isolipidic diets containing 15%, 10%, 5%, or 0% (DM basis) of WC and 2%, 3%, 4%, or 5% of CSFA as lipid sources^†^

Item	Treatments				SEM	*P*-value		
	15WC	10WC	5WC	0WC		0WC vs. WC	L	Q
Ultrasound measurements								
REA, cm^2^								
Day 0	48.8	48.5	48.5	48.1	1.62	0.77	0.75	0.97
Day 55	56.6	57.3	58.0	56.8	0.68	0.54	0.69	0.18
Day 103	67.2	67.6	67.6	66.0	0.89	0.16	0.37	0.25
Δ	18.6	19.1	19.1	17.6	0.92	0.20	0.44	0.27
Daily gain	0.181	0.186	0.185	0.171	0.009	0.20	0.44	0.27
Longissimus BFT, mm								
Day 0	2.0	2.0	2.0	1.9	0.08	0.70	0.97	0.58
Day 55	3.6	3.9	3.6	3.5	0.16	0.45	0.5	0.32
Day 103	4.9	5.3	4.9	5.1	0.27	0.79	0.76	0.65
Δ	2.9	3.4	3.0	3.2	0.28	0.79	0.76	0.64
Daily gain	0.028	0.033	0.029	0.031	0.003	0.79	0.76	0.64
Biceps femoris BFT, mm								
Day 0	2.9	3.0	3.0	3.0	0.11	0.94	0.75	0.41
Day 55	4.9	5.5	5.4	5.6	0.19	0.23	0.04	0.32
Day 103	6.6	7.2	6.7	7.0	0.28	0.57	0.49	0.51
Δ	3.6	4.3	3.8	4.1	0.28	0.59	0.54	0.52
Daily gain	0.035	0.042	0.037	0.040	0.003	0.59	0.54	0.52
Marbling, %								
Day 0	2.43	2.52	2.20	2.34	0.15	0.82	0.41	0.88
Day 55	2.94	3.11	3.01	3.25	0.07	< 0.01	0.01	0.63
Day 103	3.06	3.21	3.16	3.35	0.07	0.01	0.01	0.72
Δ	0.70	0.76	0.83	0.98	0.08	0.02	0.01	0.61
Carcass traits								
HCW, kg	259.6	260.8	258.0	267.5	9.54	0.48	0.63	0.67
DP, %	54.69	54.28	53.88	54.12	0.31	0.65	0.14	0.30
Carcass ADG, kg	1.05	1.05	1.02	1.00	0.03	0.48	0.40	0.83
YG, %	62.2	61.5	60.4	61.5	0.90	0.87	0.45	0.31

L, linear effect; Q, quadratic effect.

^†^Diets were offered for 108 d.

Lastly, no contrast effects were observed for HCW (*P* ≥ 0.48), DP (*P* ≥ 0.14), carcass ADG (*P* ≥ 0.40), or YG (*P* ≥ 0.31; Table 8).

### Meat Traits and FA Profile

No treatment effects were observed on meat pH (*P* ≥ 0.37), cooking loss (*P* ≥ 0.18), and WBSF (*P* ≥ 0.41; Table 9). For the chemical composition, a linear effect was observed on ash content (*P* = 0.04), but no further differences were detected (*P* ≥ 0.13; Table 9). Similarly, a* index tended to be reduced by including WC in the diets (*P* = 0.08) and no further differences were observed for L* and b*index (*P* ≥ 0.13; Table 9).

**Table 9.** Meat characteristics of *Bos indicus* bulls offered isolipidic diets containing 15%, 10%, 5%, or 0% (DM basis) of WC and 2%, 3%, 4%, or 5% of CSFA as lipid sources^†^

Item	Treatments				SEM	*P*-value		
	15WC	10WC	5WC	0WC		0WC vs. WC	L	Q
pH	6.11	6.22	6.23	6.24	0.10	0.63	0.37	0.64
Chemical composition, %								
Moisture	75.8	75.7	75.4	75.7	0.32	0.78	0.57	0.38
CP	21.4	21.5	21.4	21.1	0.16	0.14	0.24	0.34
EE	1.9	1.2	1.6	1.6	0.29	0.92	0.62	0.33
Ash	1.1	1.1	1.1	1.1	0.01	0.36	0.04	0.13
Colorimetric parameters^‡^								
L^*^	34.8	34.6	35.9	35.6	0.61	0.44	0.18	0.99
a^*^	19.1	19.2	19.2	19.8	0.32	0.08	0.13	0.43
b^*^	5.9	5.7	6.0	6.0	0.44	0.90	0.84	0.88
Cooking loss, %	27.1	24.7	23.8	26.8	1.96	0.49	0.84	0.18
WBSF, kg	4.9	4.6	4.8	4.4	0.59	0.96	0.41	0.46

L, linear effect; Q, quadratic effect.

^†^Diets were offered for 108 d.

^‡^L* = lightness index (0 = black and 100 = white); a* = intensity of red color, an index that ranges from green (−) to red (+); b* = intensity of yellow color, an index ranging from blue (−) to yellow (+; Houben et al., 2000).

As expected, several meat FAs were impacted by the different inclusion levels of WC and CSFA (Table 10). Inclusion of WC in the diets reduced the amount of meat C18:2 *cis*-7, *trans-*9 conjugated linoleic acid (CLA) (*P* = 0.03); C18:3 *cis*-9, *cis*-12, *cis*-15 (*P* < 0.001); C22:2 (*P* < 0.001); and *n*-3 PUFA (*P* = 0.04; Table 10). Moreover, as WC level in the diet increased, meat C16:1 *trans*-9 (*P* = 0.01); C18:2 *cis*-7, *trans-*9 CLA (*P* = 0.04); and MUFA (*P* = 0.04) content decreased, whereas C18:1 *cis*-9 tended to decrease (*P* = 0.09) and C18:0 (*P* = 0.05) and SFA (*P* = 0.03) increased (Table 10). Additional quadratic effects were observed for meat levels of C14:0 (*P* = 0.05); C16:0 (*P* = 0.01); C18:3 *cis*-9, *cis*-12, *cis*-15 (*P* = 0.01); and SFA (*P* = 0.02; Table 10). Animals fed 15WC had greater C14:0, C16:0, and saturated FA, whereas 0WC cohorts had greater meat C18:3 *cis*-9, *cis*-12, *cis*-15 (Table 10).

**Table 10.** Meat FA profile of *Bos indicus* bulls offered isolipidic diets containing 15%, 10%, 5%, or 0% (DM basis) of WC and 2%, 3%, 4%, or 5% of CSFA as lipid sources^†^

FA, % total	Treatments				SEM	*P*-value		
	15WC	10WC	5WC	0WC		0WC vs. WC	L	Q
C10:0 (capric acid)	0.07	0.07	0.08	0.07	0.01	0.69	0.55	0.31
C12:0 (lauric acid)	0.10	0.08	0.10	0.08	0.01	0.27	0.31	0.75
C14:0 (myristic acid)	3.77	3.13	3.35	3.40	0.16	0.92	0.20	0.05
C14:1 *cis*-9 (tetradecenoic acid)	0.62	0.61	0.87	0.72	0.09	0.87	0.18	0.42
C15:0 (pentadecanoic acid)	0.33	0.29	0.28	0.27	0.02	0.33	0.09	0.43
C15:1 *cis*-10 (pentadecenoic acid)	2.17	2.13	2.37	2.77	0.35	0.18	0.19	0.53
C16:0 (palmitic acid)	27.89	25.97	26.37	27.59	0.52	0.15	0.82	0.01
C16:1 *trans-*9 (palmitoleic acid)	2.02	2.41	2.89	2.93	0.22	0.12	0.01	0.44
C17:0 (heptadecanoic acid)	0.63	0.67	0.59	0.58	0.04	0.22	0.19	0.50
C17:1 *cis-*10 (heptadecenoic acid)	0.41	0.53	0.46	0.47	0.05	0.99	0.58	0.24
C18:0 (stearic acid)	14.68	13.18	12.22	12.18	0.84	0.27	0.05	0.40
C18:1 *cis*-9 (oleic acid)	29.06	35.37	33.37	34.57	1.79	0.37	0.09	0.18
C18:1 *trans*-9 (elaidic acid)	3.14	3.56	3.29	3.87	0.31	0.12	0.17	0.81
C18:2 *cis*-9, *trans*-11 CLA	9.40	8.78	7.49	7.46	1.11	0.39	0.16	0.79
C18:2 *cis*-7, *trans-*9 CLA	0.10	0.11	0.12	0.18	0.02	0.03	0.04	0.39
C18:2 *cis*-10, *trans*-10 CLA	9.48	8.86	7.58	7.63	1.12	0.43	0.19	0.77
C18:3 *cis*-9, *cis*-12, *cis*-15 (linolenic acid)	0.32	0.29	0.24	0.44	0.04	< 0.001	0.11	0.01
C20:0 (arachidic acid)	0.08	0.08	0.07	0.08	0.01	0.27	0.92	0.26
C20:1 (eicosenoic acid)	0.14	0.20	0.18	0.17	0.01	0.60	0.45	0.02
C20:2 (eicosadienoic acid)	0.17	0.16	0.15	0.17	0.03	0.87	0.83	0.66
C20:3 *n*-6 (dihomo-gamma-linolenic acid)	0.17	0.31	0.35	0.25	0.05	0.64	0.24	0.03
C20:4 (arachidonic acid)	1.42	1.31	1.43	1.30	0.22	0.72	0.80	0.95
C20:5 (eicosapentaenoic acid)	0.27	0.27	0.29	0.31	0.05	0.59	0.59	0.82
C21:0 (heneicosylic acid)	0.01	0.01	0.02	0.01	0.01	0.31	0.02	0.40
C22:0 (behenic acid)	0.02	0.01	0.01	0.01	0.01	0.1	< 0.01	< 0.01
C22:1 (erucic acid)	0.02	0.01	0.01	0.01	0.01	0.06	0.03	0.53
C22:2 (docosadienoic acid)	0.16	0.16	0.15	0.23	0.01	< 0.001	< 0.01	0.01
C23:0 (tricosylic acid)	0.12	0.11	0.10	0.12	0.02	0.70	0.90	0.55
Saturated FA	47.35	43.62	43.03	44.43	1.02	0.81	0.03	0.02
Monounsaturated FA	37.93	44.82	45.23	45.02	2.11	0.36	0.04	0.10
Polyunsaturated FA	11.96	11.46	10.23	11.70	1.25	0.74	0.72	0.45
*n*-3	0.36	0.35	0.31	0.47	0.06	0.04	0.23	0.13
*n*-6	9.36	8.77	7.49	8.45	1.05	0.94	0.36	0.45
Total FA	97.2	99.9	98.5	101.2	1.46	0.75	0.02	0.36
Ratio								
SFA:MUFA	1.25	0.97	0.95	0.99	0.07	0.13	0.04	0.02
SFA:PUFA	3.96	3.81	4.21	3.80	0.10	0.05	0.45	0.03

L, linear effect; Q, quadratic effect.

^†^Diets were offered for 108 d.

## Discussion

The main objective of the present experiment was to evaluate the effects of different levels of WC and CSFA on feedlot performance, nutrient intake, and serum concentrations of hormone and metabolites involved in lipid metabolism as well as carcass and meat characteristics of *B. indicus* bulls offered a high-concentrate diet during the finishing phase. This goal arised from the hypothesis that the association of a feedstuff with physical lipid protection (WC) and another with chemical lipid barrier (CSFA) against rumen biohydrogenation (Cappellozza et al., 2020) would allow a greater increase in dietary EE content, without impairments on rumen metabolism and microorganism population, which, in turn, would benefit performance and carcass traits of feedlot animals during the finishing phase. Moreover, the FA profile of these feedstuffs, when added in different dietary levels, would have a positive effect on marbling and BFT, improving customer eating experience and meat acceptability (Westerling and Hedrick, 1979). Therefore, based on this rationale, diets were formulated to be isocaloric, isofiber, isolipidic, and isonitrogenous (Table 3), taking into account the high-quality nutrient supply of WC, including NDF, CP, and EE (Pires et al., 1997; Mena et al., 2004; Cranston et al., 2006), and the great amount of EE from the CSFA source. This experimental design allowed the direct comparison of how the inclusion of these lipid feedstuffs (WC and CSFA) would impact performance and carcass traits of feedlot *B. indicus* beef cattle. It is noteworthy mentioning that one might question the lack of an experimental group containing only WC as the sole lipid source, but the explanation for this includes 1) possible negative effects of a greater dietary WC inclusion (>15% DM) on performance and carcass traits have been reported by others (Zinn, 1996; Cranston et al., 2006; Gouvêa et al., 2020), 2) the WC levels used herein are representative of the current nutritional practices in Brazilian feedlots (Pinto and Millen, 2019), and 3) to the best of our knowledge, no other research evaluated different levels of both lipid sources in the same experiment (Carvalho et al., 2020; Costa et al., 2020), and further studies are warranted.

The lack of differences in DMI, nutrient intake, final BW, ADG, and carcass traits might be explained by the similar nutritional content of the experimental diets. Recently, Carvalho et al. (2020) demonstrated that CSFA inclusion into a high-concentrate diet containing cottonseed byproducts (WC and cottonseed meal) yielded similar final BW and ADG, improved FE, and reduced DMI when compared with diets that did not contain CSFA. In another study, Costa et al. (2020) did not report differences in feedlot performance of *B. indicus* animals offered an isocaloric, isonitrogenous, and isolipidic high-concentrate diet containing a combination of WC and corn germ, CSFA alone, or a three-way combination of WC, corn germ, and CSFA as lipid feedstuffs. An increased linear inclusion of WC impacted DMI and performance (ADG and FE) of beef cattle, but positive effects were observed on carcass traits when WC inclusion varied from 2.2% to 10.3% DM (Gouvêa et al., 2020). The differences between the previous and the present study might be attributed to the FA profile of the supplements used (Nascimento et al., 2020) and the EE content of the diets, so that greater dietary EE levels lead to a greater reduction in DMI (Carvalho et al., 2020; Gouvêa et al., 2020), whereas the lack of differences herein might be attributed to the fact that diets were formulated to contain similar levels of energy, protein, and fat. The fact that FE was reduced in 0WC was surprising, contrary to previous studies reporting either an improvement (Carvalho et al., 2020; Nascimento et al., 2020) or similar (Costa et al., 2020) FE vs. non-CSFA-supplemented cohorts. Although formulated to be isofiber, physically effective NDF content of 0WC was 1% to 5% lower than the other treatments (Table 2). Additionally, not only the amount but also the type of fiber was different (corn silage vs. WC) between treatments, which might be able to impact rumen fermentation parameters and nutrient utilization. Supporting this rationale, the linter present in WC has a high ruminal fermentation rate that consequently improves DM degradation (Bo et al., 2012) and nutrient utilization when compared with corn silage. Recently, de Souza et al. (2018) reported differences in nutrient intake and performance of dairy cows receiving either WC or soybean hulls in the diet. More specifically, WC inclusion increased NDF, total FA, and NE for lactation intake in isolipidic and roughly isofiber diets, without reported differences on the overall DMI.

The estimated FA intake observed herein likely reflects the DMI and nutrient composition of the experimental diets, whereas ruminal biohydrogenation, blood concentrations of each FA, and orts FA were not evaluated herein. Animals that did not receive WC had an estimated greater C16:0; C18:0; C18:1 *cis*-9; C18:3 *cis*-9, *cis*-12, *cis*-15; total SFA; and MUFA intake, as well as greater SFA:PUFA ratio, and the opposite was observed when WC was included into the diets for C18:2 *cis*-9, *cis*-12 and overall PUFA. In dairy cattle, the effects of C18:0 and C18:1 *cis*-9 on metabolism and productive responses of dairy cattle receiving soybean hulls or WC have been reported by de Souza et al. (2018). Overall, supplementing C16:0 + C18:0 FA improved DMI and milk fat and protein content vs. a mixture of C16:0 and C18:1 *cis*-9 FA, whereas opposite results were observed on nutrient digestibility and BW changes. In beef cattle, few studies have evaluated the effects of these specific FA on metabolism and performance of the herd (Costa et al., 2020; Nascimento et al., 2020). Increasing the intake of C16:0 and C18:1 *cis*-9 FA improved FE, HCW, and deposition efficiency of energy of feedlot cattle when compared with animals receiving a soybean oil-based CSFA, without differences in DM and the overall nutrient intake (Nascimento et al., 2020).

Dry matter intake fluctuation might be used as an important parameter evaluating the profitability of the feedlot, given that the costs associated with feedstuffs are the greatest factor influencing the profitability of a commercial beef cattle operation, accounting for over 63% of the variation in total annual costs (Miller et al., 2001). Moreover, reducing DMI fluctuation will likely improve the predictability of feed intake by feedlot animals, resulting in a reduced amount of feed wasted in the bunk, positively affecting the profitability of the operation, and finally, likely preventing the occurrence of digestive disorders. As reported by others (Owens et al., 1998; Krehbiel, 2014), a greater variation in total DMI is commonly associated with low ruminal pH of cattle fed high-grain diets. To the best of our knowledge, this is the first research study reporting a reduced DMI fluctuation in feedlot animals receiving CSFA, and further studies are warranted to understand if this variable is impacted in other trials with different management, dietary nutritional profile, and cattle breed.

Following this rationale, the evaluation of a feed selectivity index might be useful in predicting rumen health and potential disorders, such as subacute rumen acidosis (SARA; Keunen et al., 2002; Maulfair et al., 2013). In dairy cattle, Kmicikewycz and Heinrichs (2015) reported that cows are able to change their particle size preference depending on the rumen health. More specifically, dairy cows consuming a short particle size corn silage total mixed ration (TMR) changed their preference to longer forage particles during a SARA challenge. Cows consuming long corn silage-based diets, however, selected against longer particles during the same challenge. Changes were also observed in the present study, so that a greater WC content of the diet reduced the preference for long and medium particles, while the preference for fine particles increased. To the best of our knowledge, this is one of the first experiments evaluating potential dietary interactions with feed sorting and preference in beef cattle offered a high-energy and high-lipid feedlot diet, whereas several researchers evaluated this matter in dairy cattle offered a TMR (Kmicikewycz and Heinrichs, 2015; Miller-Cushon and DeVries, 2017). The reason for these results is unknown and could be related to potential differences in rumen metabolism and health which, unfortunately, were not evaluated herein and warrant further investigation.

Circulating leptin concentration is regulated by body fat content, nutrient intake, carcass composition, and circulating insulin (Houseknecht et al., 1998; Geary et al., 2003). In agreement, Cappellozza et al. (2014) demonstrated that supplemented beef heifers with greater ADG also had greater plasma insulin and leptin concentrations when compared with unsupplemented beef heifers, which, in turn, had a reduced ADG. In the present experiment, 10WC bulls had a greater serum leptin concentration vs. other treatments on day 103 of the study (Fig. 1). These results were unexpected, considering the overall similar performance and nutrient profile of the diets. Nonetheless, 10WC animals had, numerically, a greater ADG and DMI in the last half of the experiment (day 56 to 108), which might explain the observed leptin results. Besides a greater leptin concentration, carcass traits and final carcass measurements, such as REA and BFT of longissimus and biceps femoris, did not differ among treatments (Table 8). Nonetheless, removing WC from the diets improved marbling in measurements obtained on days 55 and 103 of the study. In a previous research from the same group (Costa et al., 2020), marbling was not affected by supplementing different sources of FA to feedlot *B. indicus* animals.

In ruminants, marbling content is impacted by the dietary strategies offered to the herd, so that ingredients that increase propionate production result in a greater glycogenic and insulinogenic capacity, leading to an increased intramuscular fat deposition (Gilbert et al., 2003). However, this hypothesis might not be applicable in the present experiment, as estimated starch intake was similar between treatments. Nonetheless, it is important to mention that many of the genes that control adipocyte differentiation in cattle are regulated by FA circulating in plasma (Choi et al., 2015). Oleic acid has been recognized as a potent FA in stimulating lipid synthesis from glucose in intramuscular tissue and lipogenesis from acetate (Smith and Johnson, 2016). Hence, a tendency was observed for meat concentration of C18:1 *cis*-9 FA to increase as WC content of the diet decreased. In a previous study, Costa et al. (2020) reported that genes involved in lipid metabolism were downregulated as C16:0 and C18:1 *cis*-9 FAs intake also decreased. As an example, peroxisome proliferator-activated receptor-γ (PPAR-γ) has been a gene of interest when looking at lipid metabolism and its potential effects on meat characteristics, such as marbling (Lim et al., 2011). Another key enzyme involved in lipid metabolism is stearoyl CoA-desaturase (SCD), in a manner that its greater expression has been associated with adipocytes hypertrophy (Martin et al., 1999). Increased C18:1 *cis*-9 FA intake and ruminal FA biohydrogenation with subsequent CLA formation result in a downregulation of tissue SCD (Keating et al., 2005; Smith and Johnson, 2016). In the present experiment, as rumen FA biohydrogenation, blood circulating FA, and FA content in the orts were not measured, it could be speculated that although a greater estimated C18:1 *cis*-9 FA intake was observed in 0WC, rumen biohydrogenation likely played a role in forming intermediates CLA isomers, inhibiting enzymes involved in lipid metabolism and marbling.

In the present experiment, no treatment effects were observed for carcass pH, chemical composition, and colorimetric parameters (Table 9). In agreement with our results, others (Gilbert et al., 2003; Costa et al., 2020; Nascimento et al., 2020) also did not report effects of lipid sources and FA profile of supplements on cooking loss, carcass chemical composition, and WBSF. Conversely, Nascimento et al. (2020) observed treatments effects on b* index, in a manner that greater values were observed in *B. indicus* animals offered the same CSFA source as used herein vs. cohorts fed a CSFA source based on soybean oil. It is reported that steaks with WBSF values greater than 4.6 kg/cm^2^ are tough (Belew et al., 2003), and our results suggest that animals, regardless of treatment, had WBSF values closer to this threshold. The fact that *B. indicus* cattle was used herein might explain the lack of treatment effects on pH and shear force. Sant’Anna et al. (2019) also reported greater pH values in Nellore carcass, which might be related to the temperament and, consequently, the stress-related responses elicited by this trait (Fordyce et al., 1988; Voisinet et al., 1997). Based on these, it is logical to speculate that other factors beyond nutrition, such as cattle temperament, breed, castration status, and age, might play a role in consistently and permanently affecting carcass characteristics of *B. indicus* cattle, whereas the specific effects of CSFA, if any, are yet to be determined.

One of the major goals in ruminant nutrition is to alter the FA profile of edible products, such as beef and milk, in order to benefit the human population that consumes such products (Scollan et al., 2006; Hess et al., 2008; Jenkins et al., 2008). A feasible alternative to achieve this goal would be to feed CSFA, reducing the amount of FA being hydrogenated in the rumen (Cappellozza et al., 2020) and increasing the amount of FA reaching the small intestine for further absorption. Following the design of the experimental diets and the FA intake data, it would be speculated dramatic differences in meat FA profile, such as alterations in hypercholesterolemic FA (C14:0 and C16:0; Farfan, 1996). Indeed, feeding CSFA without WC increased meat concentration of C18:2 *cis*-7, *trans*-9; C18:3; and *n*-3 FA and reduced SFA:PUFA ratio. On the other hand, 10WC-fed animals had lower meat C14:0 and C16:0 but greater C20:1, C20:3, and total MUFA. From a human nutrition and health standpoint, a greater supply of C14:0, C16:0, and other SFA to ruminants would not be desirable, as these are widely known as hypercholesterolemic FAs (Scollan et al., 2006; Oliveira et al., 2011). More specifically, myristic FA has a 4-fold greater potential to cause an increase in blood cholesterol when compared with palmitic FA (Oliveira et al., 2012). Nonetheless, ruminants have the ability to modify palmitic FA through elongation and desaturation of the FA chain, giving rise to stearic and oleic FA, respectively (Archibeque et al., 2005; Scholljegerdes et al., 2007), which might explain some of the FA differences observed herein. On the other hand, supplementation with CSFA of a mixture of palm, soybean, and cottonseed oil yielded greater meat total PUFA when compared with meat obtained from animals receiving other treatments, which are part of the hypocholesterolemic FA class and act on cattle metabolism by reducing blood concentrations of low-density lipoproteins and increasing high-density lipoproteins, which are known to prevent the occurrence of cardiovascular disease (Molkentin, 2000).

In summary, feeding WC from 5% to 15% of dietary DM increased FE during feedlot, whereas CSFA supplementation reduced DMI fluctuation and improved marbling in *B. indicus* beef cattle offered isonitrogenous, isolipidic, and isocaloric high-concentrate diets, demonstrating the potential of CSFA to minimize health issues in the feedlot and improve carcass traits and customer eating experience. However, it remains to be evaluated possible interactions, if any, among fiber source and type on productive and metabolic responses of *B. indicus* cattle offered a finishing diet.
